# Public funding and private investment for R&D: a survey in China’s pharmaceutical industry

**DOI:** 10.1186/1478-4505-12-27

**Published:** 2014-06-13

**Authors:** Lan Qiu, Zi-Ya Chen, Deng-Yu Lu, Hao Hu, Yi-Tao Wang

**Affiliations:** 1 State Key Laboratory of Quality Research in Chinese Medicine, Institute of Chinese Medical Sciences, Block 3–406, University of Macau, Av. Padre Tomás Pereira Taipa, Macao, China; 2 International Business Faculty, Beijing Normal University, Jinfeng Road, Tangjia Wan, Zhuhai, Guangdong 519085, China

**Keywords:** China, Pharmaceutical industry, Private investment, Public funding, Research and development

## Abstract

**Background:**

In recent years, China has experienced tremendous growth in its pharmaceutical industry. Both the Chinese government and private investors are motivated to invest into pharmaceutical research and development (R&D). However, studies regarding the different behaviors of public and private investment in pharmaceutical R&D are scarce. Therefore, this paper aims to investigate the current situation of public funding and private investment into Chinese pharmaceutical R&D.

**Methods:**

The primary data used in the research were obtained from the China High-tech Industry Statistics Yearbook (2002–2012) and China Statistical Yearbook of Science and Technology (2002–2012). We analyzed public funding and private investment in five aspects: total investment in the industry, funding sources of the whole industry, differences between provinces, difference in subsectors, and private equity/venture capital investment.

**Results:**

The vast majority of R&D investment was from private sources. There is a significantly positive correlation between public funding and private investment in different provinces of China. However, public funding was likely to be invested into less developed provinces with abundant natural herbal resources. Compared with the chemical medicine subsector, traditional Chinese medicine and biopharmaceutical subsectors obtained more public funding. Further, the effect of the government was focused on private equity and venture capital investment although private fund is the mainstream of this type of investment.

**Conclusions:**

Public funding and private investment play different but complementary roles in pharmaceutical R&D in China. While being less than private investment, public funding shows its significance in R&D investment. With rapid growth of the industry, the pharmaceutical R&D investment in China is expected to increase steadily from both public and private sources.

## Background

In recent years, China has experienced a rapid growth in its pharmaceutical industry. In 2012, the pharmaceutical market in China increased by 12% to reach about 154 billion USD, and is expected to reach $383 billion by 2020 [1], making China one of the largest pharmaceutical markets globally. Meanwhile, a shift to more advanced pharmaceutical research and development (R&D) has started out in China recently. The Chinese government is dedicated to making more considerable investments in new drug R&D. Typically, in the *12*^*th*^*Five-Year Plan*, the Key Drug Innovation Program initiated by the government will receive 10,000 million Chinese Yuan (CNY) (about 1,653 million USD according to the exchange rate of 6.05 at the end of 2013) from the central government and 30,000 million CNY (about 4,959 million USD) from local governments [2]. Private investors are also motivated to invest huge funding into pharmaceutical R&D. As a result, R&D investment in the Chinese pharmaceutical industry has sharply increased [3].

However, studies regarding the different behaviors of public and private investment in pharmaceutical R&D are scarce. Since China is such a large emerging country, funding source distribution in different provinces might vary widely, as well as in different subsectors. Further, as an industry significantly driven by private venture funds, the current status of private equity (PE) and venture capital (VC) in Chinese pharmaceutical R&D investment also remains unclear.

Therefore, this paper aims to investigate the current situation of public funding and private investment in the Chinese pharmaceutical R&D. In particular, an exploration of public funding and private investment in different provinces and subsectors can help us to observe the diverse preferences of public and private investment in pharmaceutical R&D, to better understand government guidance for the Chinese pharmaceutical industry, and to identify investment opportunities and patterns in different regions and subsectors.

### Theoretical review

Recently, the Chinese government has published a series of policies to promote new drug innovation, outlining several objectives which can be considered as a systematic development strategy of China’s new drug innovation: to provide support to establish advanced-level platforms for drug discovery and development; to discover new molecular entities, new biologics (first-in-class and best-in-class) and new drugs with independent intellectual property (IP) rights; to improve modern Chinese medicine system; and to develop prospective technologies for drug R&D [2]. While such types of plan demand a joint effort of public funding and private investment, how to appropriately position two types of funding sources still requires further theoretical exploration.

Theoretically, in order to guarantee the public use of knowledge, which is the basis of applied development research, public funding is considered to be effective for basic research to generate knowledge into the form of public products [4-7]. However, knowledge from applied research and development is exclusive to some extent. Enforcement of an IP system could bring exclusiveness. For instance, patent laws could prevent the public usage of new knowledge. Private funds can then be injected in these fields [6].

Previous research on the relationship between government R&D funding and private R&D investment has not yet reached consensus. At the micro level, Link [8] found that public investment would decrease basic research of enterprises, but promote applied and development research. Hamberg [9] thought that subsidies from the United States’ Department of Defense would increase R&D investment of enterprises. At the macro level, Guellec and Van Pottlesberghe [10] have systematically studied the effect of public investment on R&D action of enterprises in 17 OECD countries from 1981 to 1996. They drew an inverted U-shaped line to describe the relationship between financial support and tax preferences from the government and the increase of private R&D investment. Toole [11] found strong evidence that public basic and clinical research were complementary to pharmaceutical R&D investment and thereby stimulated private industry investment by using a panel of medical classes observed over 18 years.

In China, Xu and Liu [12] believed the positive effect of public R&D investment, but Liu and Du [13] found that *ex ante* subsidy of the government would not improve the effort of enterprises’ innovation. Sun and Sun [14] found that government investment in research institutions did not significantly affect the R&D expenditures of pharmaceutical companies. Gu and Shen [15] found that governance environment has a positive effect on corporate R&D activities. Companies tend to carry out more R&D activities if government interference and the space of rent-seeking are reduced. Zhao [16] carried out an empirical study of Guangdong Province on the relationship between R&D investment and innovation performance, concluding that the government’s R&D investment had relevance to the level of innovation.

It is worth mentioning that David [7] collected 32 papers in this field from 1957; 20 of the papers confirmed the positive relation between public and private investment, 8 agreed that there was a complement effect between them, and 4 did not find a significant relationship. In summary, the literature has not reached a clear conclusion on the relationship between public funding and private investment for R&D in the pharmaceutical industry, especially under the changing situation that China is currently experiencing.

## Methods

### Data sources

Data was collected from multiple sources in this study. The primary data used was obtained from the China High-tech Industry Statistics Yearbook (from 2002 to 2012) and China Statistical Yearbook of Science and Technology (from 2002 to 2012), both of which were provided by the National Bureau of Statistics of China. The statistical data in these two sources covered all state-owned and non-state-owned enterprises with annual sales revenue above 5 million CNY (about 0.769 million USD). As these enterprises represented the main industrial components, it is credible to reflect the main condition and progress of R&D investment in China’s pharmaceutical industry.

Data of PE and VC in China was collected from a series of reports from ChinaVenture and Zero2IPO, both of which are integrated service providers specializing in providing professional information, data, and consulting services for PE and VC in China. In addition, comparative data of high-tech industry in other countries was obtained from the OECD, STAN Database 2011.

### Definitions

According to McGuire’s definition in 2007, the pharmaceutical industry develops, produces, and markets drugs or pharmaceuticals which are licensed for use as medications [17]. Here, we adopted the formal definition of Statistics Catalogue of High-technology Industry Classifications from National Bureau of Statistics of China, in which pharmaceutical industry only means manufacturing of medicine (code 27); the manufacturing of medical equipment and instrument (code 368) is not included into pharmaceutical industry [18].

For R&D investment, it was defined as actual internal expenses on R&D, including direct expenditure on R&D projects and indirect expenditure on management, service, basic construction, and cooperation accessing. Since R&D intensity is the most frequently used measurement “*to gauge the relative importance of R&D across industries and among firms in the same industry*” [19], we used R&D intensity as the ratio of the industry’s gross expenditure on R&D compared to the gross industrial output value.

For funding sources, we define funds from government department at all levels as public funding, and other funding sources as private investment.

### Data analysis

In order to clarify the complicated relationships between public funding and private investment in China’s pharmaceutical industry, we analyzed the data in five aspects: total investment in the industry*,* funding sources of the whole industry, difference in provinces, difference in subsectors, and PE/VC investment.

First, total investment in the industry was described by total R&D expenditure and R&D intensity. To get a better understanding of R&D investment level in China’s pharmaceutical industry, we also compared R&D intensity of several high technology industries in China with those in other selected countries.

Second, funding sources of the whole industry directly compared the two different sources of funding for R&D in the industry: public funding and private investment.

Third, differences between provinces were assessed by comparing public R&D investment intensity and private R&D investment intensity in different provinces of China. Here, the Spearman correlation test was applied to test the correlation between public R&D intensity and private R&D intensity.

Fourth, for difference in subsectors, China’s pharmaceutical industry was divided into three subsectors: chemical medicine, traditional Chinese medicine (TCM), and biopharmaceutical medicine. Potential funding differences in these three subsectors were analyzed by comparison.

Finally, PE/VC investment was analyzed as a special supplement. We collected data of PE/VC investment amount and number of cases in recent years and listed the typical cases to highlight those high-tech driven investments.

All statistical calculations were performed with the SPSS software version 18, and Excel 2003. Figures in CNY were transformed to USD value by using exchange rates in 2013 unless otherwise specified.

## Results

### Total investment in the industry

**Figure 1:**
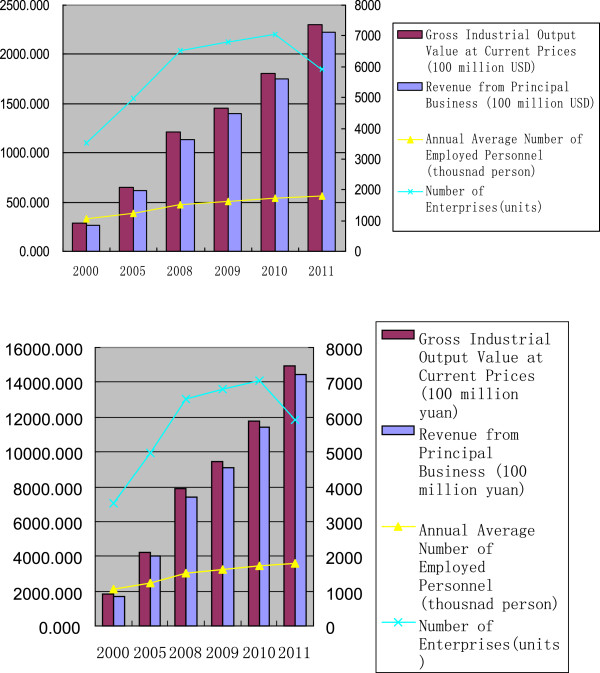
Recent changes in the Chinese pharmaceutical industry.

As China’s overall economy has grown rapidly over the past years, its pharmaceutical industry has also experienced a surge. The gross output value of the Chinese pharmaceutical industry grew from 22.26 billion USD in 2000 to 229.88 billion USD in 2011. The revenue and profit of the industry has also expanded by nearly 10 times during this period, although the number of enterprises decreased dramatically in 2011 from over 7,000 to 5,926 (Figure 1).

As the Chinese pharmaceutical industry begins to target higher-end drug products, domestic investment into the pharmaceutical innovation has largely increased. The personnel involved in Scientific and Technologic (S&T) activities increased to nearly 119,000 in 2011, whereas the volume of patent applications went up by 19.32 times from 2000 to 2011 (Table 1). There was a decrease in both personnel for S&T and patent application numbers in 2010, which may be partially explained by a stricter regulation resulting from China’s new health care reform effective in that year.

**Table 1:** Emerging R&D activities in the Chinese pharmaceutical industry

	**2000**	**2005**	**2007**	**2008**	**2009**	**2010**	**2011**
Personnel for scientific and technologic (S&T) activities	37,833	51,832	73,408	90,820	90,222	70,781	118,558
Patent applications	547	2,708	3,056	3,917	8,601	5,767	11,115
R&D expenditure (100 million CNY)	13.46 (163 million USD)	39.95 (494 million USD)	65.90 (889 million USD)	79.10 (1155 million USD)	135.00 (2045 million USD)	122.60 (1851 million USD)	211.20 (3,352 million USD)

Source: China High-tech Industry Statistics Yearbook.

**Figure 2:**
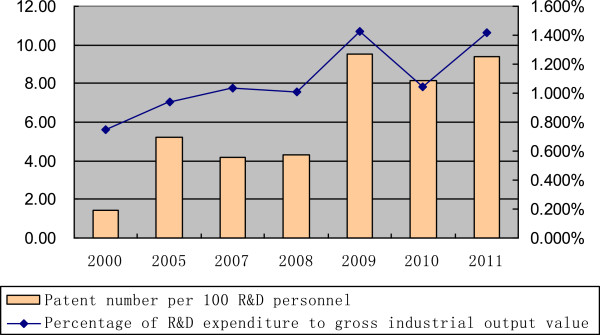
R&D activity of the Chinese pharmaceutical industry in recent years.

The R&D expenditure in China’s pharmaceutical industry has increased from 162.6 million USD in 2000 to 3,249.2 million USD in 2011. The R&D intensity also fluctuated in 2010 and rose back to 1.41% in 2011 (Table 1 and Figure 2). However, it is far less than that of some other high-tech industries in China, such as the aircraft and spacecraft industry, with a ratio of 7.82%.

Furthermore, the R&D intensity of the pharmaceutical industry in the United States and UK in 2007 was 26.57% and 24.92% respectively, which shows a tremendous gap between China and other developed economies in pharmaceutical R&D investment (Table 2).

**Table 2:** The ratio of R&D expenditure to gross industrial output value of high-tech industries in selected countries

	**High technology industry**	**Aircraft and spacecraft**	**Pharmaceuticals**	**Office, accounting and computing machinery**	**Radio, television and communication equipment**	**Medical, precision and optical instruments**
China (2011)	1.63	7.82	1.41	0.75	1.81	1.91
USA (2007)	16.89	9.90	26.57	10.69	15.72	18.34
Japan (2008)	10.50	2.90	16.40	7.61	8.90	16.98
Germany (2007)	6.87	8.65	8.27	4.46	6.28	6.28
UK (2006)	11.10	10.70	24.92	0.38	7.56	3.63
France (2006)	7.74	5.20	8.69	7.94	12.24	7.08
Italy (2007)	3.82	13.43	1.79	1.23	4.48	2.60
Canada (2006)	11.50	6.27	11.88	10.92	14.52	–
Spain (2007)	5.22	6.87	6.25	3.80	3.85	3.24
Korea (2006)	5.86	9.02	2.51	3.93	6.65	2.16
Sweden (2007)	13.18	12.91	13.44	13.92	14.73	8.99
Denmark (2006)	–	–	18.40	5.09	11.49	8.32
Norway (2007)	5.67	1.09	5.48	0.85	7.51	5.91
Finland (2007)	11.50	4.81	24.50	2.34	11.76	4.91

Source: China High-tech Industry Statistics Yearbook.

### Funding sources of the whole industry

**Figure 3:**
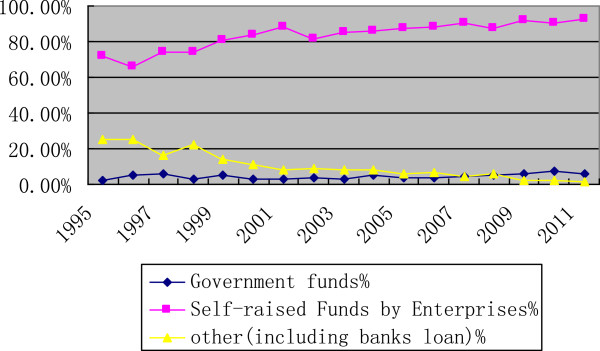
Change of R&D sources in the Chinese pharmaceutical industry.

The majority of R&D investment was from self-raised funds of pharmaceutical enterprises in this R&D booming period. The percentage of private investment in the overall R&D investment was 71.5% in 1995 and increased to 92.73% in 2011. Meanwhile, the percentage of government funds rose slightly from 2.5% in 1995 to 6.03% in 2011. Other sources decreased obviously from more than 20% to 1.24% over the same period (Figure 3).

### Difference in provinces

**Figure 4:**
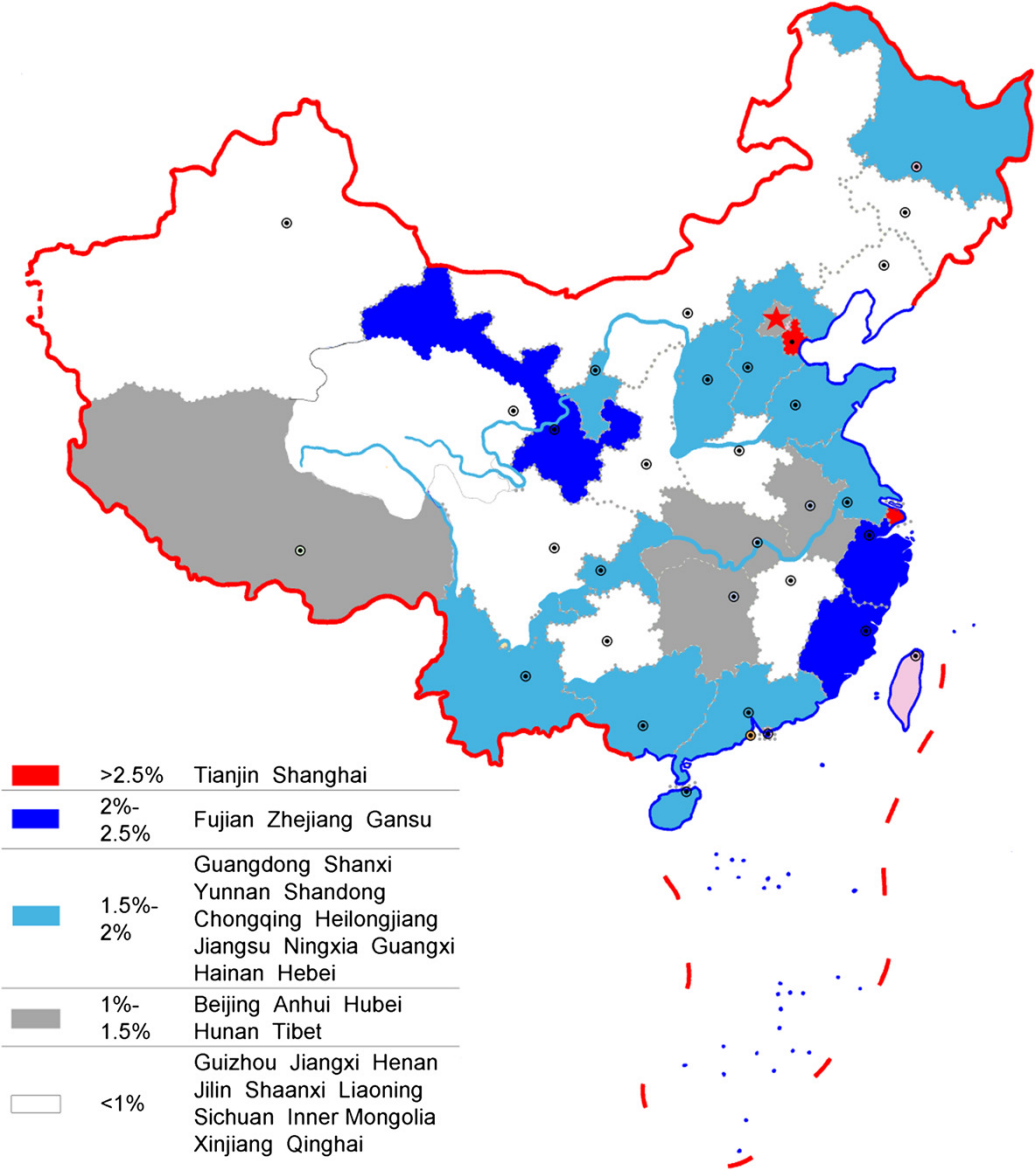
Provincial imbalance of R&D intensity in pharmaceutical industry.

The map of provincial R&D intensity in 2011 presented a regional imbalance of R&D investment (Figure 4). The R&D intensity of the eastern region of China was 1.88%, which is higher than the average ratio of 1.41%, and it was 0.839% and 0.778%, respectively, for the middle and western regions of China.

There were 17 provinces or municipality cities with an above average ratio, from which two municipality cities – Tianjin with 2.987% and Shanghai with 2.963% – were the two highest. It is a little surprising that Fujian, Zhejiang, and Gansu followed closely, as opposed to those provinces known to have a large-scale pharmaceutical industry such as Shandong or Jiangsu (see navy blue areas in Figure 4). Shandong was the sixth and Jiangsu ranked outside the top 10 provinces.

However, according to the ratio of investment from government to output value (Table 3), the ranking was a little different. Gansu jumped to the first, nearly twice of the second, Tianjin. Gansu is a typical province where pharmaceutical industry development is mainly promoted by the local government based on its rich Chinese herbal resources, although it is one of the least developed provinces in China. The export volume of its Chinese angelica, *Codonopsis pilosula*, and *Astragalus mongholicus* accounted for more than 80% of China’s total amount. Recently, the high volume of funding has been invested in the modernization of TCM R&D in Gansu.

**Table 3:** Provinces with highest ratio of public investment to output value

**Rank**	**Province**	**Ratio**
1	Gansu	0.003644
2	Tianjin	0.0019844
3	Guangdong	0.0019062
4	Hainan	0.0018739
5	Chongqing	0.0017726
6	Yunnan	0.001762
7	Hubei	0.0015216
8	Fujian	0.0014481
9	Beijing	0.0014014
10	Guizhou	0.0013565

Source: China High-tech Industry Statistics Yearbook; China Statistical Yearbook.

Guangdong, Hainan, and Chongqing ranked behind Tianjin. Guangdong, ranking as the fourth in gross pharmaceutical industrial output value in China, injected both public and private resources into the industry. Shanghai, with a developed non-public economic sector, decreased to the eleventh. Zhejiang, which is famous for its small enterprises and free-market environment, was listed as fourteenth.

**Figure 5:**
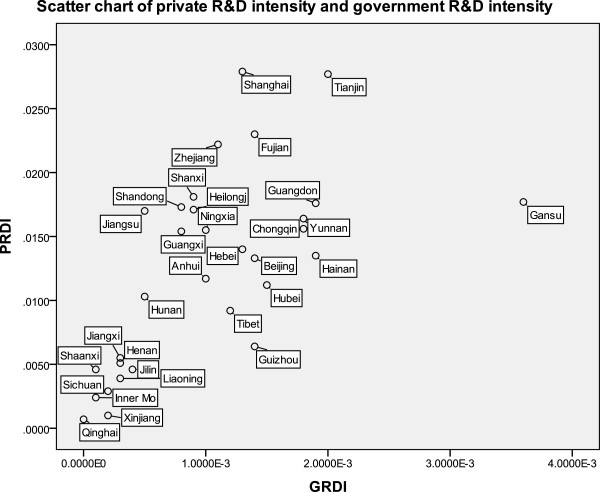
Scatter diagram of public and private R&D intensity in different provinces.

The Spearman correlation test showed that the correlation coefficient between private R&D intensity and government R&D intensity was 0.689 (Table 4). The scatter figure also showed their positive correlation; however, Gansu was slightly distinctive because of its high government R&D investment intensity compared with its private R&D investment intensity (Figure 5).

**Table 4:** Spearman correlation test for public and private R&D intensity

			**Private R&D intensity**	**Government R&D intensity**
Rho of Spearman	Private R&D intensity	Correlation coefficient	1.000	0.689**
		Sig. (2 tailed)	0	0
		N	31	31
	Government R&D intensity	Correlation coefficient	0.689**	1.000
		Sig. (2 tailed)	0	0
		N	31	31

**P <0.01.

### Difference in subsectors

**Figure 6:**
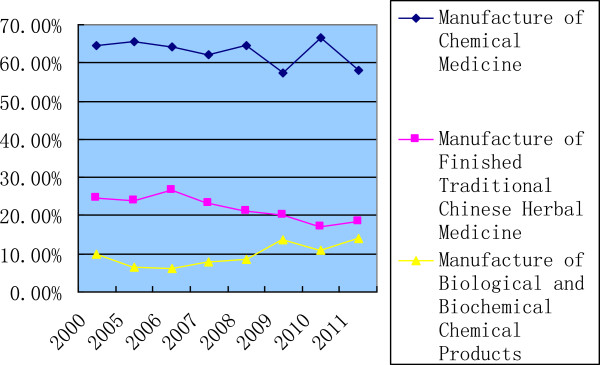
Percentage change of R&D investment in three subsectors.

In 2011, 57.99% of R&D investment, totaling 1.9 billion USD, was put into the chemical medicine subsector, the TCM subsector received 18.32% or 0.6 billion USD, and the biopharmaceutical subsector attracted 0.45 billion USD, accounting to 13.89%. The percentage of R&D investment in chemical medicines had kept above 60% during most years from 2000 to 2013, but the ratio of TCM decreased from 24.5% in 2000 and the biopharmaceutical subsector attracted more and more funds during this period (Figure 6).

**Figure 7:**
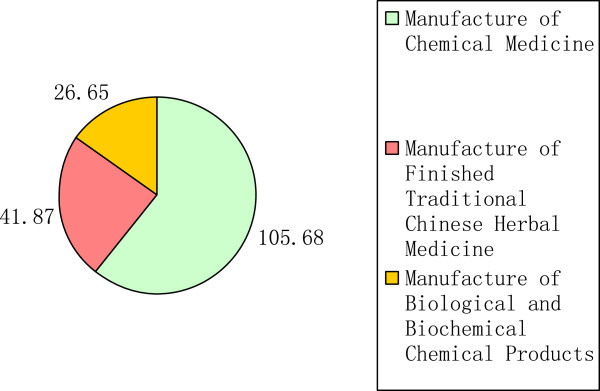
Proportion of public R&D investment in three subsectors in 2011.

In 2011, public investment in chemical medicines reached 105.7 million USD, being much higher than the sum of that in TCM (41.87 million USD) and biopharmaceuticals (26.65 million USD) (Figure 7).

Table 5 shows the scale of public investment in three subsectors. The fact that most governmental resources had been invested into the chemical medicine subsector has not changed in the past decade, and may be related to the large scale and number of chemical medicine firms in China. However, the biopharmaceutical subsector experienced the fastest growth; the public investment in the biopharmaceutical subsector increased by more than 13 times from 2000 to 2011, while the remaining two subsectors increased by 9.5 and 8.4 times, respectively.

**Table 5:** Scale of public R&D investment in three subsectors (10,000 CNY)

	**2000**	**2005**	**2006**	**2007**	**2008**	**2009**	**2010**	**2011**
Chemical Medicine	7,263 (8.77 million USD)	17,216 (21.3 million USD)	21,644 (27.63 million USD)	27,454 (37.03 million USD)	36,415 (53.16 million USD)	38,947 (57.04 million USD)	51,276 (77.42 million USD)	68,693 (109.04 million USD)
TCM	3,235 (3.91 million USD)	10,436 (12.92 million USD)	10,650 (13.6 million USD)	17,758 (23.95 million USD)	21,764 (31.77 million USD)	22,780 (33.36 million USD)	16,824 (25.4 million USD)	27,218 (43.2 million USD)
Biopharmaceutical	1,318 (1.59 million USD)	4,789 (5.93 million USD)	4,418 (5.64 million USD)	7,127 (9.61 million USD)	7,572 (11.05 million USD)	14,551 (21.31 million USD)	16,840 (25.43 million USD)	17,322 (27.5 million USD)

Source: China High-tech Industry Statistics Yearbook.

**Figure 8:**
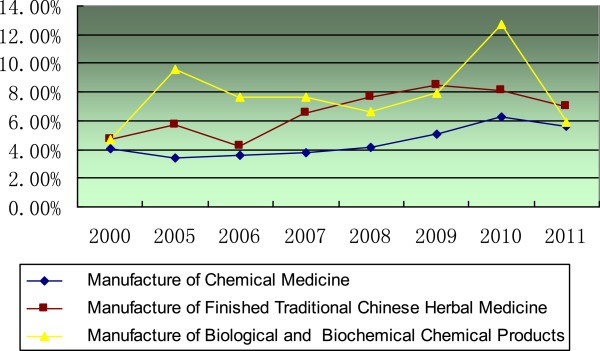
Percentage change of public funding in three subsectors.

More details about percentage of public R&D investment in total R&D investment showed that the percentage of public investment in the chemical medicine subsector had kept on around 5%, and the percentage of public investment in the biopharmaceutical subsector was the highest but not stable. The percentage of public R&D investment in all three subsectors increased before 2009 and decreased in 2011 (Figure 8).

### PE/VC investment

**Figure 9:**
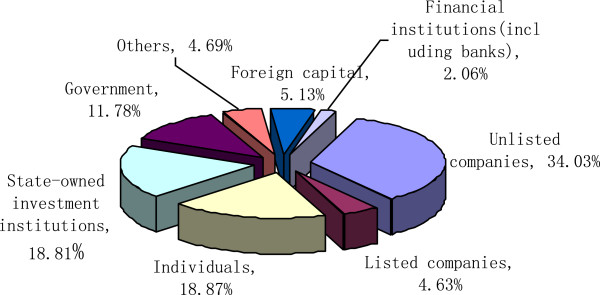
Capital sources of Chinas venture capital.

Similar with most countries, private investment was the main source of China’s PE/VC. In 2012, unlisted companies accounted for 34.03% of the total VC sources and individual sources accounted for 18.87% [20]. The government and state-owned enterprises together accounted for 30.59% (Figure 9).

It is worth mentioning that there is a VC Guiding Fund (VCGF) which had made a cumulative investment of 28.9 billion CNY (4.44 billion USD) by the end of 2012. These investments operated by governments have been injected into VC by buying VCs shares, risk subsidies, and investment guarantee. In 2012, 5% of investment of VCGF-backed VC has been input in pharmaceutical and healthcare industry.

**Figure 10:**
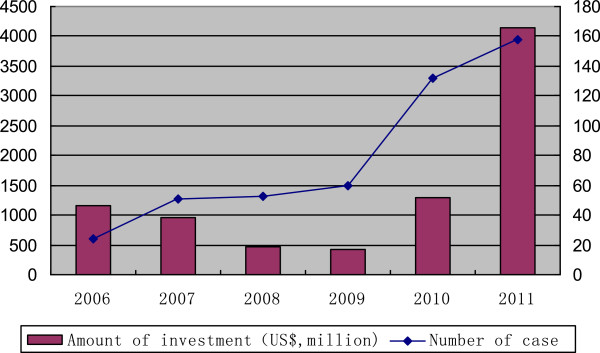
Booming PE/VC in Chinese pharmaceutical industry.

According to Zero2IPO Research Center, in 2012, China’s PE market completed 680 investment deals, of which 606 deals disclosed 19.79 billion USD in total. Among these deals, biopharmaceutical investment held the majority. Figure 10 presents the increase of PE/VC investment deals and amounts in China in the past several years. There was a decline in 2008 and 2009 when the world was still struggling to shake off the impact of financial crisis, but the situation improved and the PE/VC investment hit a new high in 2011.

PE investment cases in 2012 are listed in Table 6, showing that companies focusing on a particular high-tech field or a particular market segment, though in a small scale, were preferred by institutional investors. For example, *Luye Pharma Group Ltd*. is well known by its successful TCM innovation and long-acting sustained-release microsphere preparation R&D, while *Gansu Longshen Rongfa Pharmaceutical Co., Ltd*. engages mainly in the cutting-edge research of TCM.

**Table 6:** PE investment cases in 2012

**Enterprises**	**Amount**	**Investor**	**Issue time**
Nantong Novast Pharmaceuticals Co., Ltd.	20 million USD	Lilly Asia Ventures	13/6/2012
Gansu Longshen Rongfa Pharmaceutical Co., Ltd.	2.9 million USD	Genertec Investment Managers Ltd.	29/3/2012
Luye Pharma Group Ltd	N/A	CDH Investments; CITIC Private Equity Funds Management Co., Ltd.; New Horizon	3/2/2012
Shaanxi Bicon Pharmaceutical Co., Ltd.	250 million USD	Pacific Alliance Group	28/2/2012
Kaifeng Pharmaceutical (Group) Co., Ltd.	14.7 million USD	N/A	20/2/2012

Source: ChinaVenture.

## Discussion

Overall, we have observed a rapid increase of investment in Chinese pharmaceutical R&D during the past decade, although the investment intensity is still behind other high-tech industries around the world. The results indicate that China still needs more pharmaceutical R&D investment to realize its objective of developing a competent pharmaceutical innovation system.

### Private investment as a main funding source

As our data showed, private investment was the main funding source for pharmaceutical R&D in China. It is understandable for private investors to choose to invest in applied research and development research; however, if the increasing public input cannot fully support basic research and early stage R&D activities, the Chinese pharmaceutical industry will not be able to reach high levels until after many years’ effort. Further, drug R&D activity and mass production, especially for first-in-class drugs, are time-consuming [21,22]. This may hinder private investors, who are seeking short-term return, from injecting their money in early drug R&D stages. Meanwhile, in China, what puzzles institution investors mostly is how to withdraw capital gains because of strict initial public offering regulations and an underdeveloped merger/acquisition market.

For private investors, the best solution is to increase the firm’s profit expectation of R&D. Firms with a significant IP stake in China could put in place an effective protection and enforcement strategy against local and overseas competitors [23]. Practical and effective IP protection and enforcement strategies should be an integrated part of a firm’s overall strategy. Mortgage on IP has also been undertaken in some cities in China and could have great potentials, although problems, such as valuation and liquidation of IP, have not yet been resolved.

### Differences of public and private R&D investment in provinces

Many studies have concluded that pharmaceutical firms prefer to cluster around developed areas with sufficient talents and massive funds [24-26]. However, in many countries, industry agglomeration is usually led by both the government and market; the same is true in China.

Gansu Province ranked the first in the public R&D investment intensity list, mainly because of its large volume of herbal resources. However, due to its remote location from the eastern and southern economy centers of China, the scale of its whole economy and pharmaceutical industry is very limited. The gross industrial output value of Gansu Province in 2012 was 958 million USD, ranking fourth last among all provinces. It is obvious that the main objective of public funding should be to support industrial development in less developed areas.

It is not surprising that Tianjin ranked almost the highest in both the lists of public R&D investment intensity and total R&D investment intensity. Since *Binhai New Area* in Tianjin was established as a new administrative division in 2009, the local government strongly promoted scientific, technological, and financial innovations and took great efforts to establish science and technology-based financial conglomerates [27]. As a result, investment on high-tech industry, including the biopharmaceutical industry, has been high in that region. It ranked as the best investment area in China in 2010.

Shanghai, with its *Zhangjiang Hi-Tech Park* being already the largest new drug R&D base in China, has attracted relatively more investment from enterprises, including many multinational pharmaceutical companies like Novartis, GlaxoSmithKline, and Pfizer. More than 210 pharmaceutical companies and dozens of drug R&D institutes or centers have been established in Shanghai.

In general, the provinces with high R&D investment intensity are mainly distributed in eastern and southern China. Both the scatter chart and Spearman test show us an obvious positive relationship between public funding and private investment. The possible situation is a mutual promotion: local governments in provinces with developed private economies tend to inject more capital in R&D and have ability to do so, on the contrary, private investments are guided and attracted by favored government funding policies.

Besides the developed districts like Tianjin, Shanghai, and Guangdong Province, half of the top 10 provinces with the highest public investment intensity are developing areas with abundant natural resources, such as Gansu, Yunnan, Hainan, and Guizhou Provinces. Governments in these provinces lean towards developing the TCM industry by favored policies and public funding, which also promote the investment from private investors.

### Differences of public and private R&D investment in subsectors

The chemical medicines subsector is the largest in China’s pharmaceutical industry, and China is the largest exporter of active pharmaceutical ingredients [28]. Accordingly, we have observed a constant and large volume of R&D investment into this subsector, being the subsector with the largest public investment. However, if the percentage of public investment in the three subsectors is taken into account, the highest percentage of investment was observed in the biopharmaceutical subsector before 2007, and TCM became the highest between 2007 and 2009. After 2009, the biopharmaceutical was once again the highest.

The percentage change of public R&D investment in the three subsectors partially reflects government intentions. A large problem in the current pharmaceutical industry in China is the over-capacity of active pharmaceutical ingredients and chemical drugs without high technical innovation [29]. Therefore, the overall profits of the pharmaceutical industry have been decreasing; a new driving force is required to push the industry.

The shift from pharmaceutical development towards discovery research may originate from two considerations. The first is to deepen the R&D of TCM, which has not been widely accepted in western countries. In recent years, the percentage of public R&D investment in the TCM subsector reached 8%. Meanwhile, a list of development strategies of TCM has been released by the Chinese government. Another signal is that the new Essential Drug List was promulgated in early 2013, including 203 TCMs among 520 drugs in total. There are 40 exclusive TCM products, and each of them is authorized to be produced by only one TCM firm [30]. It is a policy sign to attract more attention and investment on modernization of TCM.

The second is to seize the growth opportunity of biopharmaceutical S&T. Successive and increasing investments, an especially high percentage of public R&D investments, reflect the potential growth of the biopharmaceutical subsector in China. Many policy makers in China believe that the biopharmaceutical field is one in which Chinese pharmaceutical firms can become world leaders. Despite the impassable gulf between Chinese firms and enterprises in developed countries in the field of chemical medicines, the short history of China in the biopharmaceutical field opens the possibilities of it becoming world leader. Both governmental and private sources have been attracted to this field; high-tech companies, which take advantage of genetic engineering technologies to develop genomic drugs, are favored by Chinese private investors.

### PE and VC in China’s pharmaceutical industry

In 1998, a proposal to Develop Venture Capital Investment in China by the National Committee of the Chinese People’s Political Consultative Conference was considered to be a trigger to attract VC investment for high-tech industries. Particularly, in the stock exchange market, the opening of the Small and Medium Enterprises Board in 2004 and the Growth Enterprises Market Board in 2009, stimulated the acceleration of PE and VC. Besides, a series of government policies, as well as Company Law and Securities Law, have been promulgated to promote investor entry, market access, and capital exit for PE and VC.

Currently, in China, both case number and amount of PE and VC investment are in a rising channel. As data shows, the proportion of individuals and foreign capital increased significantly, mainly because the Chinese government encourages foreign capital to invest in high-tech industries. It also led to significant increases of individual investors as angel investors.

Although the vast majority of investment amount of VC is from private sources, government inflcuence is still of significance. For example, the most important information channel of VC investment projects is government recommendation, which has kept on more than 25% among all information channels in the past 5 years. According to a survey in 2012, 18.9% of VC investors thought the main reason for unsuccessful investment was policy change in the policy environment [31]. Specific to investment behavior, VCGF-backed VC is more likely to invest in medicine and healthcare industry, and to select projects in a mature stage [31].

The greatest difficulty for PE and VC in China is the lack of an efficient investment exit channel. As a result, many PE and VC focus on short-term and less innovative projects. More investment exit channels will effectively promote PE and VC investors to seek more long-term and creative projects, which will certainly benefit the innovation of pharmaceutical R&D in China.

## Conclusions

Public funding and private investment play different but complementary roles in the pharmaceutical R&D of China. Public funding tends to be injected into some undeveloped provinces, and biopharmaceutical and TCM subsectors. While being less than private investment, public funding shows its significance in R&D investment. With rapid industry growth, the pharmaceutical R&D investment in China is expected to keep a constant increase from both public and private sources.

## Abbreviations

CNY: Chinese Yuan; IP: Intellectual property; PE: Private equity; R&D: Research and development; S&T: Scientific and Technologic; TCM: Traditional Chinese medicine; VC: Venture capital; VCGF: VC Guiding Fund.

## Competing interests

The authors declare that they have no competing interests.

## Authors’ contributions

LQ designed the study, conducted data collection and analysis, and drafted the manuscript. ZYC and DYL participated in data collection. HH participated in research design and manuscript revision. YTW reviewed the whole manuscript. All the authors read and approved the final manuscript.
